# Impact of Carotid Artery Geometry and Clinical Risk Factors on Carotid Atherosclerotic Plaque Prevalence

**DOI:** 10.3390/jpm15040152

**Published:** 2025-04-12

**Authors:** Dac Hong An Ngo, Seung Bae Hwang, Hyo Sung Kwak

**Affiliations:** 1 Department of Radiology, Research Institute of Clinical Medicine, Jeonbuk National University, Biomedical Research Institute of Jeonbuk National University Hospital, 20, Geonji-ro, Deokjin-gu, Jeonju-si 54907, Jeollabuk-do, Republic of Korea; ngodachongan@hueuni.edu.vn (D.H.A.N.); sbh1010@jbnu.ac.kr (S.B.H.); 2 Department of Radiology, University of Medicine and Pharmacy, Hue University, Hue 530000, Vietnam

**Keywords:** carotid artery, geometry, atherosclerosis, plaque, computed tomography angiography

## Abstract

**Objectives:** Carotid geometry and cardiovascular risk factors play a significant role in the development of carotid atherosclerotic plaques. This study aimed to investigate the correlation between carotid plaque formation and carotid artery geometry characteristics. **Methods:** A retrospective cross-sectional analysis was performed on 1227 patients, categorized into a normal group (n = 685) and carotid plaque groups causing either mild stenosis (<50% stenosis based on NASCET criteria, n = 385) or moderate-to-severe stenosis (>50%, n = 232). The left and right carotid were evaluated individually for each group. Patient data, including cardiovascular risk factors and laboratory test results, were collected. Carotid geometric measurements were obtained from 3D models reconstructed from cranio-cervical computed tomography angiography (CTA) using semi-automated software (MIMICS). The geometric variables analyzed included the vascular diameter and sectional area of the common carotid artery (CCA), internal carotid artery (ICA), external carotid artery (ECA), and carotid artery bifurcation (CAB), as well as the carotid bifurcation angles and carotid tortuosity. **Results:** Compared to the normal group, in both the right and left carotid arteries, patients with carotid plaques exhibited a significantly higher age (*p* < 0.001) and a greater prevalence of hypertension (*p* < 0.001) and diabetes mellitus (*p* < 0.001). Additionally, they demonstrated a larger CCA and a smaller carotid bifurcation dimension (*p* < 0.05). In the analysis of the left carotid artery, patients with carotid plaques also had a significantly smaller ICA dimension (*p* < 0.05) than the normal group. **Conclusions:** This study found that patients with carotid plaques were older and had a higher prevalence of hypertension and diabetes, larger CCAs, and smaller carotid bifurcations. The plaque-positive left ICA was significantly smaller than that of the plaque-negative group, suggesting a side-specific vulnerability. These findings highlight the role of carotid geometry in plaque formation and its potential clinical implications for personalized risk assessment and targeted interventions.

## 1. Introduction

Atherosclerosis is a leading cause of cardiovascular diseases, significantly contributing to morbidity and mortality worldwide [1]. Among its most severe clinical manifestations, stroke remains a major health concern, often resulting in long-term disability and death [2]. Carotid artery disease, primarily due to atherosclerotic plaque formation, accounts for approximately 7% to 25% of ischemic strokes [3,4], underscoring the critical role of carotid atherosclerosis in cerebrovascular events.

The formation of atherosclerosis is a chronic inflammatory process within the arterial wall, initiated by the entry and accumulation of cholesterol-rich apo-B lipoproteins, such as low-density lipoprotein cholesterol (LDL-C), into the subendothelial space [5]. This accumulation triggers an inflammatory response, recruiting macrophages and T cells that interact with arterial wall cells, with inflammation caused by obesity potentially accelerating this process through the release of various cytokines from adipose tissue and activated macrophages [6]. Furthermore, perivascular adipose tissue, an active endocrine organ surrounding blood vessels, can secrete adipokines, cytokines, and growth factors that either inhibit or stimulate cardiovascular disease (CVD) development, and its dysfunction can lead to inflammation and oxidative stress [7]. Endothelial dysfunction also plays a critical role, with obesity impairing communication between endothelial cells and leading to abnormal vasodilation due to increased reactive oxygen species (ROS) and disrupted signaling [8]; additionally, endothelial damage can be induced by microparticles released from cells, particularly under high glucose conditions, exacerbating inflammation. A key feature of atherosclerosis progression involves vascular smooth muscle cells (VSMCs), which exhibit phenotypic plasticity and can transition from a contractile to a synthetic state, increasing proliferation and extracellular matrix remodeling; importantly, VSMCs are also central to vascular calcification—the deposition of hydroxyapatite in both the intimal and medial layers of arteries—driven by active reprogramming of VSMCs in response to local environmental cues, such as inflammation, oxidative stress, and apoptosis in atherosclerotic calcification [9]. Moreover, in the context of prediabetes, which can precede the onset of diabetic symptoms and is associated with an increased risk of atherosclerosis, extracellular vesicles and microRNAs have been found to be involved in the progression of atherosclerosis, with hyperglycemia facilitating the production of extracellular vesicles that carry specific proteins like ROS-producing NADPH oxidase and LDL-scavenging CD36, as well as microRNAs that promote inflammation and hematopoiesis [6]. This intricate interplay of lipid deposition, inflammation, endothelial dysfunction, VSMC modulation, and calcification contributes to the characteristic structural and histological changes of atherosclerosis [9].

Carotid atherosclerosis progresses through distinct molecular stages, beginning with lipid accumulation and endothelial dysfunction, where oxidized LDL-C triggers inflammation and immune cell recruitment [10]. Macrophage polarization into pro-inflammatory and anti-inflammatory states influences plaque stability, with pro-inflammatory-dominated plaques being more vulnerable [11]. Ferroptosis, extracellular matrix degradation, and dysregulated lipid metabolism contribute to necrotic core expansion and fibrous cap thinning. As the disease advances, neoangiogenesis and intraplaque hemorrhage promote plaque instability, while microcalcifications and oxidative stress further weaken structural integrity [12,13]. Ultimately, plaque rupture exposes thrombogenic material, leading to atherothrombosis and ischemic events [14]. Carotid geometry changes that lead to areas with fluctuating flow influence plaque formation and vulnerability, highlighting the complex interplay between hemodynamics and molecular pathology [15].

The development of atherosclerotic plaques within the carotid artery is not random but predominantly occurs at specific anatomical sites, particularly at the carotid bifurcation and the lateral wall of the internal carotid artery (ICA) [16,17]. Hemodynamic factors, such as wall shear stress (WSS), play a crucial role in plaque formation and progression. Regions of low and oscillatory WSS have been associated with endothelial dysfunction, increased low-density lipoprotein uptake, and subsequent atherosclerotic plaque accumulation [16,17]. Conversely, areas subjected to high WSS and laminar flow may exhibit a protective effect against plaque formation [16,17]. Carotid hemodynamics is strongly associated with anatomical characteristics. Moreover, carotid geometry has been shown to significantly influence hemodynamic patterns [18], thereby affecting the development of carotid plaques. Despite being exposed to the same systemic risk factors, carotid plaques do not develop symmetrically within an individual. The research of Larson et al. showed that plaques on the left side are more frequently associated with unstable features, making them more likely to cause an ipsilateral stroke [19]. This result suggests that carotid anatomy and geometry contribute significantly to the pathogenesis of atherosclerosis [20,21]. Several studies have proposed that variations in carotid bifurcation geometry, vessel diameter, cross-sectional area, and flow ratios influence local hemodynamics, thereby affecting plaque formation and vulnerability [22,23]. Other studies have reported strong associations between geometric parameters and plaque volume, while others suggest that these relationships are weak or inconclusive [24,25]. However, the role of carotid geometry as an independent predictor of plaque vulnerability requires further investigation, particularly in clinical populations at risk of atherosclerotic disease.

Based on this background literature, we hypothesize that carotid atherosclerotic plaques may be linked to carotid geometry and cardiovascular risk factors. Specifically, we sought to determine whether changes in the carotid artery dimensions (vessel diameter and sectional area), bifurcation angle, and tortuosity (the degree to which a vessel curves or twists) are associated with plaque presence. These findings provide insights that could aid in developing a more personalized approach to cardiovascular risk stratification and improving carotid plaque prevention.

## 2. Materials and Methods

This study was conducted in alignment with the principles of the Declaration of Helsinki and was authorized by the Institutional Review Board of Jeonbuk National University Hospital (JUH 2024-03-014, approval date: 20 March 2024).

### 2.1. Patient Demographic

The study protocol was approved by the Institutional review board of the university’s hospital. Between January 2019 and December 2022, data from 1571 patients, including their clinical information, laboratory results, and carotid computed tomography angiography (CTA) images for neurovascular disease, were collected for analysis.

A total of 152 patients were excluded due to incomplete carotid CTA examinations, and an additional 192 were excluded based on the following criteria: a history of carotid interventions (surgical or endovascular procedures), complete occlusion of the ICA, severe stenosis caused by plaques in the common carotid artery (CCA), or carotid artery torsion resulting from prior thoracic surgery or pulmonary tuberculosis.

This resulted in a final cohort of 1227 patients. Of these, 685 were classified into the normal group with no evidence of carotid plaques, while 542 patients exhibited carotid plaques on either side with varying degrees of stenosis. In patients with carotid plaques, the contralateral normal carotid artery (if present) was not used for analysis to ensure the consistency of the normal group. The left and right carotid arteries were analyzed separately. Based on the degree of stenosis, diseased carotid arteries were classified into group 1 (<50% stenosis) and group 2 (≥50% stenosis). The severity of carotid stenosis was evaluated using the NASCET (North American Symptomatic Carotid Endarterectomy Trial) criteria [26].

### 2.2. CTA Imaging Protocol

All CTA examinations were conducted using a dual-source CT system (SOMATOM Definition Flash; Siemens Healthcare, Erlangen, Germany). Initially, a non-contrast CT scan was performed from the foramen magnum to the vertex, followed by a CTA covering the region from the ascending aorta to the intracranial arteries. Iodinated contrast agent (Ultravist; Schering, Berlin, Germany) was administered at a rate of 5 mL/s, followed by a saline flush. The CTA data had 1 mm slice thickness reconstructions and 5 mm maximum intensity projection (MIP) reconstructions.

### 2.3. Carotid Artery 3D Model Reconstruction

**Figure 1:**
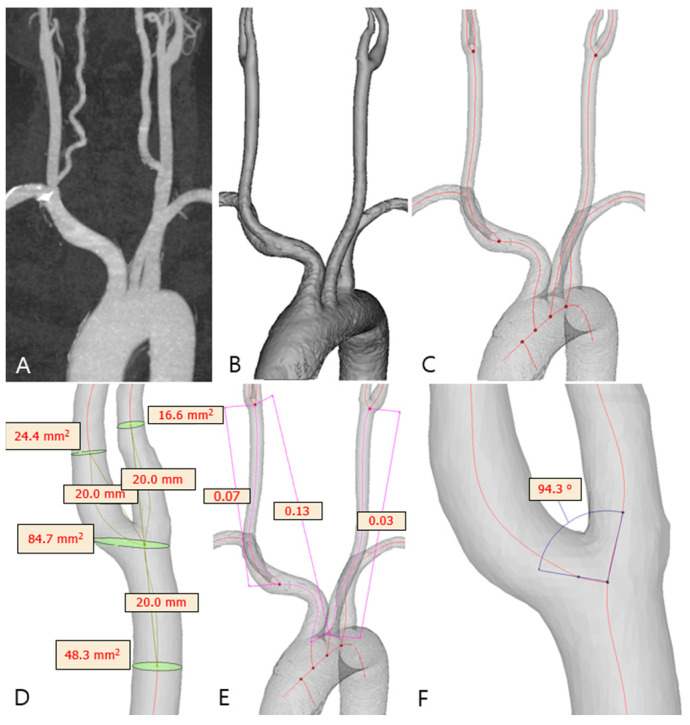
**Carotid geometry reconstruction and measurement.** (**A**) Carotid computed tomography angiography (CTA) data of a representative case used for the carotid 3D model reconstruction. (**B**) A 3D model reconstructed from the CTA data by semi-automated software. (**C**) The arterial centerline (red line) reconstructed as a 3D model. (**D**) Measurements of carotid geometry variables on the 3D model. (**E**) Measurements of carotid tortuosity. (**F**) Measurement of a carotid bifurcation angle.

The CTA images of patients (Figure 1A) were collected in the Digital Imaging and Communications in Medicine (DICOM) format and transferred to semi-automated software (Materialise MIMICS v25.0; Materialise NV, Leuven, Belgium) for 3D reconstruction. The carotid system was segmented using different methods, including thresholding, region growing, and manual segmentation, to achieve the desired model (Figure 1B). The final 3D model of the carotid system included the aortic arch, brachiocephalic trunk, bilateral CCA, carotid bifurcation, ICA, and external carotid artery (ECA). Other small branches were excluded from the model. The model was then fine-tuned for optimal resolution; the surface was smoothed to reduce noise. The centerline from the aorta to both the ICA and ECA was automatically generated as a continuous line through the centers of spheres with the maximum radius that could fit within the corresponding vessel lumen (Figure 1C).

### 2.4. Carotid Geometry Measurement

The 3D models generated from the CTA data were utilized for geometric assessments. Measurements of the common carotid artery (CCA), ICA, and ECA diameters and sectional areas (Figure 1D) were obtained on planes perpendicular to the centerline 2 cm from the carotid bifurcation point. Measurements of the carotid bifurcation (CAB) diameter and sectional area were performed on the corresponding plane containing the bifurcation point. The carotid bifurcation angle (Figure 1F) was defined as the largest angle formed by the vector projections of the ICA and ECA on the bifurcation plane. The ICA angle was measured as the angle between the CCA and ICA vectors on a plane tangential to the bifurcation plane. Vessel tortuosity (Figure 1E) was calculated as the ratio of the straight-line distance to the centerline length between the proximal and distal points of the vessel. The tortuosity index was determined for the segment extending from the brachiocephalic artery origin to the carotid bifurcation and from the CCA origin to the carotid bifurcation.

### 2.5. Other Clinical Variables

Diabetes mellitus (DM) was classified based on either a fasting glucose concentration ≥7.0 mmol/L, a non-fasting glucose concentration ≥11.1 mmol/L, diabetes management medication use, or self-reported diabetes. Hypertension was defined as a systolic blood pressure ≥140 mm Hg, a diastolic blood pressure ≥90 mm Hg, or the use of antihypertensive drugs. Dyslipidemia was identified via the use of antilipidemic medication or having one or more of the following: elevated total cholesterol ≥5.17 mmol/L (200 mg/dL); high triglycerides ≥1.70 mmol/L (150 mg/dL); a low concentration of high-density lipoprotein cholesterol (HDL-C) of ≤1.29 mmol/L (50 mg/dL) in females and ≤1.03 mmol/L (40 mg/dL) in males; and a high LDL-C of ≥3.36 mmol/L (130 mg/dL). Smoking was defined as an individual who was smoking or had stopped smoking for <5 years. Diagnosis of atrial fibrillation was based on the medical history or electrocardiogram examination upon admission or during hospitalization. Alcohol drinkers were defined as those with weekly alcohol use in the past year.

### 2.6. Statistical Analysis

Statistical analyses were performed with SPSS version 23.0 (IBM Corp., Armonk, NY, USA). For continuous variables, data were reported as mean ± standard deviation or median (interquartile range), depending on the distribution. Categorical variables were expressed as frequencies and percentage. A chi-squared test (for categorical variables) or a one-way ANOVA (for continuous variables) was performed to evaluate the mean differences of variables among the 3 groups (normal, plaque with mild stenosis (group 1), and plaque with moderate-to-severe stenosis (group 2)). Post hoc analysis was performed using the Bonferroni algorithm. The Spearman’s correlation test was used to assess the association between carotid plaque groups and their risk factors and geometry variables. Spearman’s rank correlation coefficient (rho) was used to assess the relationship between variables. A *p*-value of less than 0.05 was considered statistically significant.

## 3. Results

**Figure 2:**
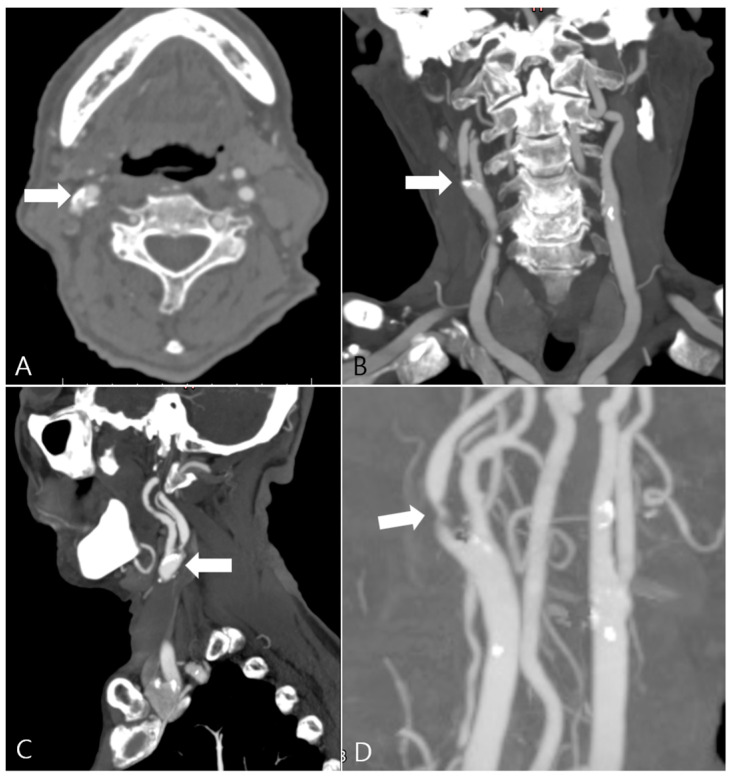
**Representative case of carotid stenosis.** Carotid computed tomography angiography (CTA) images of a representative patient with a carotid plaque causing severe stenosis in the right internal common carotid artery (arrows), visible in the axial plane (**A**), coronal plane (**B**), sagittal plane (**C**), and maximum intensity reconstruction (**D**).

Among the 1227 subjects included in the study, 685 were classified as normal, with no evidence of carotid plaques. Of the remaining patients, 190 right carotid plaques and 170 left carotid plaques were associated with mild stenosis (<50%), while 108 right carotid plaques and 99 left carotid plaques were associated with moderate stenosis (≥50%) (Figure 2). Demographic, risk factor, and carotid geometry data are summarized in Table 1, Table 2 and Table 3.

### 3.1. Analysis of Right Carotid Plaque (Table 1 and Table 2)

The mean age was significantly higher in groups 1 and 2 compared to the normal group (77.25 ± 0.66 years and 75.69 ± 0.89 years, respectively, vs. 69.99 ± 0.41 years, *p* < 0.001). Hypertension and DM were also more prevalent in groups 1 and 2 compared to the normal group (hypertension: 74.2% and 65.7%, respectively, vs. 51.5%, *p* < 0.001; DM: 32.6% and 35.2%, respectively, vs. 21.9%, *p* < 0.001).

Regarding carotid geometry, groups 1 and 2 demonstrated significantly larger CCA diameters compared to the normal group (7.65 ± 0.07 mm and 7.69 ± 0.1 mm, respectively, vs. 7.35 ± 0.03 mm, *p* < 0.001). The bifurcation diameter was smaller in groups 1 (10.38 (1.99) mm) and 2 (10.14 (3.04) mm) compared to the normal group (10.57 (2.27) mm), although statistical significance was observed only between group 2 and the normal group (*p* = 0.002). No significant differences were noted between groups 1 and 2 with regard to the bifurcation diameter.

The sectional area of the CCA was significantly larger in groups 1 and 2 compared to the normal group (44.28 (15.29) mm^2^ and 44.80 (15.98) mm^2^, respectively, vs. 41.73 (13.39) mm^2^, *p* < 0.05). For the bifurcation sectional area, groups 1 and 2 exhibited smaller values compared to the normal group, but a significant difference was identified only between group 1 and the normal group (82.09 ± 2.07 mm^2^ vs. 89.19 ± 1.11 mm^2^, *p* = 0.009).

Spearman’s correlation analysis (Table 4) showed a significant positive correlation between carotid plaque status and age (rho = 0.275, *p* < 0.001), HTN (rho = 0.172, *p* < 0.001), diabetes (rho = 0.124, *p* < 0.001), atrial fibrillation (rho = 0.067, *p* = 0.035), CCA diameter (rho = 0.133, *p* < 0.001), and CCA sectional area (rho = 0.115, *p* < 0.001). Significant negative correlations were also found between carotid plaque status and the bifurcation diameter (rho = −0.092, *p* = 0.004), and the carotid plaque status and sectional area (rho = −0.102, *p* = 0.001).

No statistically significant differences were observed in ICA geometry, carotid tortuosity, or carotid angles among the groups.

### 3.2. Analysis of Left Carotid Plaque (Table 1 and Table 3)

For the left carotid plaque analysis, similar trends were observed as with the right carotid plaque, with a few additional differences. Groups 1 and 2 exhibited a significantly higher mean age compared to the normal group (76.51 ± 0.73 years and 75.71 ± 0.87 years, respectively, vs. 69.99 ± 0.41 years, *p* < 0.001). Additionally, the prevalence of hypertension was higher in groups 1 and 2 compared to the normal group (71.2% and 69.7%, respectively, vs. 51.5%, *p* < 0.001), as was the prevalence of DM (29.4% and 35.4%, respectively, vs. 21.9%, *p* = 0.004).

In terms of carotid geometry, groups 1 and 2 had significantly larger CCA diameters compared to the normal group (7.41 ± 0.07 mm and 7.46 ± 0.1 mm, respectively, vs. 7.23 ± 0.03 mm, *p* = 0.006). Conversely, the carotid bifurcation diameter was smaller in group 1 (10.93 ± 0.15 mm) and 2 (10.4 ± 0.25 mm) compared to the normal group (11.13 ± 0.07 mm, *p* = 0.002).

Additional findings were noted regarding ICA geometry. Group 2 demonstrated a significantly smaller ICA diameter compared to the normal group (5.06 (1.32) mm vs. 5.32 (1.13) mm, *p* = 0.025), while no significant difference was observed between groups 0 and 1. A similar pattern was observed for ICA sectional area, where group 2 had a significantly smaller value compared to the normal group (21.07 ± 0.89 mm^2^ vs. 23.32 ± 0.31 mm^2^, *p* = 0.033), with no significant difference between groups 0 and 1.

Spearman’s correlation analysis (Table 4) showed a significant positive correlation between carotid plaque status and age (rho = 0.254, *p* < 0.001), HTN (rho = 0.170, *p* < 0.001), DM (rho = 0.106, *p* = 0.001), CCA diameter (rho = 0.077, *p* = 0.017), and CCA sectional area (rho = 0.071, *p* = 0.028). Significant negative correlations were also found between the carotid plaque status and the bifurcation diameter (rho = −0.106, *p* = 0.001), bifurcation sectional area (rho = −0.106, *p* = 0.001), ICA diameter (rho = −0.066, *p* = 0.042), and ICA sectional area (rho = −0.070, *p* = 0.029).

No statistically significant differences were observed among the groups with respect to vascular tortuosity or carotid angles on the left side.

## 4. Discussion

### 4.1. Carotid Plaques Are Associated with Older Age, Hypertension, and Diabetes Mellitus

Our study revealed the impact of age, hypertension, and diabetes mellitus on bilateral carotid atherosclerosis. Carotid plaque formation is strongly associated with aging, hypertension, and DM, contributing significantly to the burden of cardiovascular disease in older populations. The prevalence of carotid artery disease increases markedly with age, rising from 0.1% in men under 50 to over 3% in men over 80, with age-related carotid disease reaching up to 70% when combined with vascular risk factors [27]. More severe carotid atherosclerosis (≥50% stenosis) has been observed in 5.1% to 8.3% of elderly men and 4.3% to 7.8% of elderly women [28]. Hypertension and DM are major risk factors for carotid plaque formation, significantly increasing the odds of both the presence and progression of atherosclerosis. Individuals with carotid plaques often exhibit a higher prevalence of hypertension and DM than those without, and elevated glucose levels have been linked to increased plaque formation [29]. The presence of multiple cardiovascular comorbidities, including hypertension and DM, is one of the strongest predictors of carotid atherosclerosis. Key risk factors, such as advanced age, male sex, smoking, high blood pressure, and hyperlipidemia, further contribute to carotid atherosclerosis, with elevated lipoprotein levels being independently associated with increased plaque formation [30]. Another study has also highlighted the significant relationship between carotid intima–media thickness, lipid profile abnormalities, and cardiovascular risk factors, particularly in individuals with DM [31]. The increased carotid intima–media thickness and dyslipidemia observed in DM patients may further contribute to plaque formation and vulnerability [32].

In our research, the higher prevalence of hypertension in the mild stenosis group compared to the moderate-to-severe stenosis group may be explained by a combination of physiological and survival factors. First, as carotid stenosis becomes more severe, cerebral perfusion may decrease, triggering autoregulatory mechanisms that lead to lower systemic blood pressure, which could explain the reduced hypertension rates in the advanced stenosis group [33]. Second, there may be a survivor bias, as individuals with both severe hypertension and severe carotid stenosis are at a higher risk of fatal cardiovascular events, leading to the lower representation of such individuals in the moderate-to-severe stenosis group [34]. This aligns with findings that suggest an association between lower diastolic blood pressure and increased carotid stenosis severity, indicating that individuals with well-controlled or lower blood pressure may be more likely to progress to severe stenosis while remaining in the study population [35]. These findings suggest that the relationship between hypertension and carotid plaque progression is complex and influenced by both physiological adaptations and population-level characteristics.

Our study found a higher prevalence of atrial fibrillation (AF) in patients with carotid atherosclerotic plaques, which may be explained by shared cardiovascular risk factors such as hypertension, diabetes, and aging [36]. Both atherosclerosis and AF are linked to systemic inflammation, endothelial dysfunction, and vascular remodeling, reinforcing their close relationship [37]. However, the role of dyslipidemia in this association remains controversial. While our study did not find a significant correlation between lipid levels and carotid plaques, prior meta-analyses have reported conflicting findings. A recent meta-analysis [38] suggested that higher total cholesterol (TC) levels were inversely associated with AF risk, while triglycerides, HDL-C, and LDL-C showed no clear relationship with AF incidence. This discrepancy highlights the complexity of lipid metabolism in cardiovascular disease and suggests that factors beyond dyslipidemia, such as arterial stiffness and systemic inflammation, may play a more prominent role in AF development in patients with carotid atherosclerosis [39].

### 4.2. Carotid Plaques Are Correlated with a Smaller Carotid Bifurcation and a Larger CCA

Our study demonstrated a significant correlation between carotid plaque presence and carotid geometry, specifically showing that patients with carotid plaques tend to have smaller bifurcations and larger CCAs by sectional area and diameter. These findings align with previous research, reinforcing the critical role of vascular geometry in plaque development. The study by Jiang et al. [23] found that vulnerable plaques are associated with a smaller luminal expansion ratio, defined as the bifurcation/CCA ratio. Similarly, Gregg et al. [22] reported a negative relationship between the bifurcation flare (bifurcation/CCA) and carotid plaque volume, suggesting that as flare increases, plaque volume decreases. Together, these studies suggest that smaller bifurcations may predispose individuals to plaque formation by altering local hemodynamics. From a hemodynamic perspective, the bifurcation region is known to be susceptible to disturbed flow patterns, which contribute to atherogenesis. A smaller bifurcation, as observed in our study, can exacerbate low and oscillatory WSS, which are well-established promoters of endothelial dysfunction and atherogenesis [40]. In contrast, a larger CCA may lead to altered hemodynamics, potentially increasing flow separation at the bifurcation and enhancing the residence time of blood, further facilitating atherosclerosis. Computational fluid dynamic (CFD) models further support this theory by illustrating that regions of low WSS and high OSI correlate with plaque-prone areas [41]. Furthermore, a reduced bifurcation angle and smaller bifurcation area can create more complex flow patterns, leading to prolonged exposure to low WSS, which promotes lipid accumulation and inflammatory processes. Additionally, a larger CCA diameter can modify the velocity profile entering the bifurcation, influencing the development of turbulent or recirculatory flows that further contribute to endothelial dysfunction [42].

### 4.3. A Left-Sided Carotid Plaque Is Correlated with a Smaller ICA

Our study also highlights a significant relationship between carotid plaque presence and a smaller ICA diameter on the left side. This finding is consistent with previous research indicating that ICA narrowing is a critical factor in atherosclerosis progression. The study by Phan et al. found that a reduced ICA radius at bifurcation is associated with an increased risk of plaque development [43]. A smaller ICA diameter alters hemodynamic conditions in ways that promote plaque formation. First, increased blood flow velocity in a narrowed ICA elevates WSS, which, when excessive, can lead to endothelial injury and inflammation. Second, disturbed flow patterns and a higher OSI in a reduced ICA diameter create conditions favorable for lipid accumulation and inflammatory cell infiltration, accelerating atherogenesis. These biomechanical stresses contribute to plaque growth, increasing the overall risk of carotid artery disease and its complications.

Interestingly, our study found this significant association between ICA geometry and plaque presence only on the left carotid artery. Several hemodynamic and anatomical differences exist between the left and right carotid arterial systems, influencing plaque formation and stroke risk. The left CCA originates directly from the aortic arch, exposing it to stronger pulsatile forces, whereas the right CCA arises from the brachiocephalic trunk, which dampens flow fluctuations. The left carotid artery generally experiences greater oscillatory shear stress (OSS) and disturbed flow due to its sharper angulation and direct exposure to aortic pulsatility, making it more prone to atherosclerotic plaque formation [44]. In contrast, the right carotid artery has a smoother relative flow profile with lower OSS, which may reduce plaque accumulation. These hemodynamic differences have been confirmed in CFD studies, which demonstrate that variations in shear stress contribute to endothelial dysfunction and atherogenesis. Clinically, the increased susceptibility of the left carotid artery to plaque formation may help explain the higher incidence of left-sided strokes observed in some populations [19].

In other studies, the carotid tortuosity and bifurcation angle were found to have correlations with carotid plaques [23]. These geometry factors influence local hemodynamics, which can contribute to plaque formation by altering shear stress distribution. A larger bifurcation angle and increased tortuosity are often associated with disturbed flow patterns, including regions of low and oscillatory shear stress, which promote endothelial dysfunction and atherogenesis. However, our study found no significant correlation between these geometric factors and carotid plaques, likely due to variations in study populations, sample sizes, measurement techniques, and confounding risk factors, such as age, hypertension, and lipid levels. Additionally, plaque development is a multifactorial process influenced by systemic and genetic factors beyond local hemodynamics, which may explain the inconsistencies in our results.

The results of our study facilitate a more patient-specific approach to carotid atherosclerosis prevention and management by enabling a more precise assessment of individual risk and optimizing treatment strategies. Traditional risk assessment methods often rely on population-based metrics, which may not fully capture the influence of patient-specific vascular geometry on plaque development and progression. By incorporating detailed morphological parameters—such as the lumen diameter, bifurcation angle, and arterial tapering—along with hemodynamic analysis, clinicians can identify high-risk individuals based on their unique hemodynamic environment. This approach allows for the early detection of flow disturbances, including low wall shear stress and recirculation zones, which are known to promote plaque formation and instability [16,17]. Moreover, geometry-specific modeling can guide pharmacological treatments based on an individual’s vascular profile. By integrating patient-specific carotid geometry into risk assessment and treatment planning, this approach advances personalized medicine, ultimately improving patient outcomes and reducing the incidence of stroke and cardiovascular complications [45,46].

### 4.4. Limitations

Our study has some limitations that should be acknowledged. First, patient recruitment was conducted at a tertiary medical center, which may introduce selection bias, as these patients tend to have more comorbidities and advanced disease compared to the general population. This could affect the generalizability of our findings. Second, carotid geometric measurements relied on third-party software (MIMICS), which may limit reproducibility due to software-specific algorithms and user dependency. Future studies utilizing standardized, widely accessible measurement methods could enhance comparability across different research settings. The integration of artificial intelligence (AI) may also enhance carotid plaque evaluation by analyzing complex anatomy and imaging data and automating plaque characterization. AI models offer promising solutions for identifying symptomatic and vulnerable plaques, improving risk stratification and clinical decision-making [47].

## 5. Conclusions

In conclusion, our study highlights key factors associated with carotid plaques. We found that advanced age, hypertension, and diabetes were significantly correlated with the presence of carotid plaques. Additionally, carotid geometry, including a larger CCA and a smaller carotid bifurcation, were also associated with plaque formation. Notably, in the left carotid artery, a smaller ICA dimension was specifically linked to plaque presence. The observed differences in plaque presence and carotid geometry between the left and right arteries suggest potential hemodynamic variations between the two sides, warranting further investigation for a more comprehensive understanding of carotid plaque pathophysiology. In clinical practice, our results highlight the importance of considering both systemic risk factors and local vascular morphology in the assessment of atherosclerosis risk. The side-specific differences in carotid plaque formation may provoke clinical implications for personalized risk stratification and targeted interventions in stroke management.

## Figures and Tables

**Table 1:** Demographic and risk factor data in the normal group (no carotid plaque) and in patients with carotid plaques categorized into group 1 (plaques with mild stenosis) and group 2 (plaques with moderate-to-severe stenosis).

	Right Carotids					Left Carotids				
Variables	Total (N = 983, 100%)	Normal (N = 685, 69.7%)	Group 1 (N = 190, 19.3%)	Group 2 (N = 108, 11%)	*p*	Total (N = 954, 100%)	Normal (N = 685, 71.8%)	Group 1 (N = 170, 17.8%)	Group 2 (N = 99, 10.4%)	*p*
Age, years	72.02 ± 0.34	69.99 ± 0.41	77.25 ± 0.66	75.69 ± 0.89	**<0.001**	71.75 ± 0.35	69.99 ± 0.41	76.51 ± 0.73	75.71 ± 0.87	**<0.001**
Sex, male	451 (45.9)	310 (45.3)	78 (41.1)	63 (58.3)	0.013	449 (47.1)	310 (45.3)	74 (43.5)	65 (65.7)	**<0.001**
Alcohol	156 (15.9)	122 (17.8)	15 (7.9)	19 (17.6)	**0.004**	158 (16.6)	122 (17.8)	17 (10)	19 (19.2)	**0.038**
Smoking	109 (11.1)	81 (11.8)	22 (11.6)	6 (5.6)	0.151	107 (11.2)	81 (11.8)	20 (11.8)	6 (6.1)	0.229
HTN	565 (57.5)	353 (51.5)	141 (74.2)	71 (65.7)	**<0.001**	543 (56.9)	353 (51.5)	121 (71.2)	69 (69.7)	**<0.001**
Diabetes	250 (25.4)	150 (21.9)	62 (32.6)	38 (35.2)	**<0.001**	235 (24.6)	150 (21.9)	50 (29.4)	35 (35.4)	**0.004**
Dyslipidemia	118 (12)	75 (10.9)	26 (13.7)	17 (15.7)	0.265	106 (11.1)	75 (10.9)	19 (11.2)	12 (12.1)	0.941
Atrial fibrillation	164 (16.7)	101 (14.7)	46 (24.2)	17 (15.7)	**0.008**	155 (16.2)	101 (14.7)	41 (24.1)	13 (13.1)	**0.008**

Note: HTN: hypertension. For continuous variables, data were reported as mean ± standard deviation or median (interquartile range), depending on the distribution. For categorical variables, data were expressed as frequencies (percentage). A one-way ANOVA was performed to test the mean differences of continuous variables among the three groups. Chi-square tests were performed to assess the association between categorical variables across the three groups. Bold font indicates statistical significance.

**Table 2:** Right carotid artery geometry of the normal group (no carotid plaque) and patients with carotid plaques categorized into group 1 (mild stenosis) and group 2 (moderate-to-severe stenosis).

Variables	Total (N = 983, 100%)	Normal (N = 685, 69.7%)	Group 1 (N = 190, 19.3%)	Group 2 (N = 108, 11%)	*p*	*p* ^[1]^	*p* ^[2]^	*p* ^[3]^
Vascular Tortuosity								
Brachiocephalic to Bifurcation	0.16 (0.11)	0.16 (0.11)	0.17 (0.12)	0.15 (0.12)	0.197	0.399	>0.99	0.301
CCA to Bifurcation	0.10 (0.11)	0.10 (0.11)	0.12 (0.11)	0.09 (0.12)	0.186	0.293	>0.99	0.366
Vascular Diameter								
ICA (mm)	5.19 ± 0.03	5.17 ± 0.03	5.27 ± 0.06	5.19 ± 0.1	0.388	0.506	>0.99	>0.99
ECA (mm)	4.17 ± 0.03	4.17 ± 0.03	4.18 ± 0.06	4.14 ± 0.08	0.9	>0.99	>0.99	>0.99
CCA (mm)	7.45 ± 0.03	7.35 ± 0.03	7.65 ± 0.07	7.69 ± 0.1	**<0.001**	**<0.001**	**0.001**	>0.99
Bifurcation (mm)	10.45 (2.30)	10.57 (2.27)	10.38 (1.99)	10.14 (3.04)	**0.002**	0.31	**0.002**	0.192
Cross-Sectional Area								
ICA (mm^2^)	20.40 (9.10)	20.29 (9.21)	20.92 (9.51)	20.57 (8.73)	0.958	>0.99	>0.99	>0.99
ECA (mm^2^)	14 ± 0.17	13.97 ± 0.19	13.91 ± 0.4	14.3 ± 0.52	0.808	>0.99	>0.99	>0.99
CCA (mm^2^)	42.61 (14.10)	41.73 (13.39)	44.28 (15.29)	44.80 (15.98)	**<0.001**	**0.004**	**0.031**	>0.99
Bifurcation (mm^2^)	87.03 ± 0.93	89.19 ± 1.11	82.09 ± 2.07	82.03 ± 2.74	**0.002**	**0.009**	0.051	>0.99
ICA Angle (degree)	18.61 (17.73)	18.45 (17.77)	20.19 (17.89)	18.35 (17.95)	0.737	>0.99	>0.99	>0.99
Bifurcation Angle (degree)	39.05 ± 0.5	39.16 ± 0.6	38.9 ± 1.19	38.63 ± 1.53	0.938	>0.99	>0.99	>0.99

Note: CCA: common carotid artery, ECA: external carotid artery, ICA: internal carotid artery. For continuous variables, data were reported as mean ± standard deviation or median (interquartile range), depending on the distribution. Post hoc analysis (with the Bonferroni algorithm) was conducted to compare pairwise differences, with *p*-value ^[1]^ representing the comparison between the normal group and group 1, *p*-value ^[2]^ comparing the normal group vs. group 2, and *p*-value ^[3]^ comparing group 1 vs. group 2. Bold font indicates statistical significance (*p* < 0.05).

**Table 3:** Left carotid artery geometry in the normal group (no carotid plaque) and patients with carotid plaques categorized into group 1 (mild stenosis) and group 2 (moderate-to-severe stenosis).

Variables	Total (N = 954, 100%)	Normal (N = 685, 71.8%)	Group 1 (N = 170, 17.8%)	Group 2 (N = 99, 10.4%)	*p*	*p* ^[1]^	*p* ^[2]^	*p* ^[3]^
Vascular Tortuosity								
CCA to Bifurcation	0.10 (0.09)	0.1 (0.09)	0.11 (0.09)	0.08 (0.08)	0.084	0.502	0.397	0.08
Vascular Diameter								
ICA (mm)	5.30 (1.15)	5.32 (1.13)	5.33 (1.04)	5.06 (1.32)	**0.017**	>0.99	**0.025**	**0.022**
ECA (mm)	4.14 ± 0.03	4.13 ± 0.03	4.19 ± 0.07	4.18 ± 0.08	0.602	>0.99	>0.99	>0.99
CCA (mm)	7.29 ± 0.03	7.23 ± 0.03	7.41 ± 0.07	7.46 ± 0.1	**0.006**	**0.044**	**0.041**	> 0.99
Bifurcation (mm)	11.02 ± 0.06	11.13 ± 0.07	10.93 ± 0.15	10.4 ± 0.25	**0.002**	0.719	**0.001**	0.09
Cross-Sectional Area								
ICA (mm^2^)	23.19 ± 0.27	23.32 ± 0.31	23.88 ± 0.67	21.07 ± 0.89	**0.019**	>0.99	**0.033**	**0.021**
ECA (mm^2^)	13.56 (6.98)	13.64 (7.20)	13.50 (6.34)	13.48 (6.26)	0.872	>0.99	>0.99	>0.99
CCA (mm^2^)	42.27 ± 0.34	41.65 ± 0.39	43.51 ± 0.8	44.39 ± 1.29	**0.014**	0.125	**0.049**	> 0.99
Bifurcation (mm^2^)	92.63 ± 1	94.16 ± 1.15	91.68 ± 2.25	83.66 ± 3.75	**0.006**	> 0.99	**0.005**	0.117
ICA Angle (degree)	24.31 (22.98)	24.62 (22.45)	25.2 (25.14)	21.71 (67.86)	0.115	0.545	0.52	0.117
Bifurcation Angle (degree)	45.78 ± 0.57	45.65 ± 0.65	47.36 ± 1.55	43.95 ± 1.63	0.288	0.769	> 0.99	0.374

Note: CCA: common carotid artery, ECA: external carotid artery, ICA: internal carotid artery. For continuous variables, data were reported as mean ± standard deviation or median (interquartile range), depending on the distribution. Post hoc analysis (with the Bonferroni algorithm) was conducted to compare pairwise differences, with *p*-value ^[1]^ representing the comparison between the normal group and group 1, *p*-value ^[2]^ comparing the normal group vs. group 2, and *p*-value ^[3]^ comparing group 1 vs. group 2. Bold font indicates statistical significance (*p* < 0.05).

**Table 4:** Spearman’s correlation analysis between carotid plaque status (normal group, group 1—mild stenosis, group 2—moderate-to-severe stenosis) and other variables.

	Right Carotids		Left Carotids	
Variables	Spearman’s rho	*p*	Spearman’s rho	*p*
Age	0.275	**<0.001**	0.254	**<0.001**
Alcohol	−0.067	0.035	−0.043	0.186
Smoking	−0.044	0.170	−0.038	0.244
HTN	0.172	**<0.001**	0.170	**<0.001**
Diabetes	0.124	**<0.001**	0.106	**0.001**
Atrial Fibrillation	0.067	**0.035**	0.052	0.108
Dyslipidemia	0.051	0.107	0.009	0.773
Brachiocephalic to Bifurcation Tortuosity	<0.001	0.980	Not measured	
CCA to Bifurcation Tortuosity	0.008	0.812	−0.005	0.889
ICA Diameter (mm)	0.028	0.374	−0.066	**0.042**
ECA Diameter (mm)	−0.001	0.992	0.021	0.522
CCA Diameter (mm)	0.133	**<0.001**	0.077	**0.017**
Bifurcation Diameter (mm)	−0.092	**0.004**	−0.106	**0.001**
ICA Sectional Area (mm^2^)	−0.001	0.988	−0.070	**0.029**
ECA Sectional Area (mm^2^)	0.011	0.723	0.010	0.755
CCA Sectional Area (mm^2^)	0.115	**<0.001**	0.071	**0.028**
Bifurcation Sectional Area (mm^2^)	−0.102	**0.001**	−0.106	**0.001**
ICA Angle (degree)	Not measured		−0.022	0.500
Bifurcation Angle (degree)	−0.023	0.467	0.015	0.647

Note: CCA: common carotid artery, ECA: external carotid artery, ICA: internal carotid artery, HTN: hypertension. Spearman’s rank correlation coefficient: rho. Bold font indicates statistical significance (*p* < 0.05).

## Data Availability

The data presented in this study are available upon request from the corresponding author. The data are not publicly available due to privacy restrictions.

## Abbreviations

The following abbreviations are used in this manuscript:

AI	Artificial intelligence
CAB	Carotid artery bifurcation
CCA	Common carotid artery
CFD	Computational fluid dynamics
CTA	Computed tomography angiography
DM	Diabetes mellitus
ECA	External carotid artery
HDL-C	High-density lipoprotein cholesterol
ICA	Internal carotid artery
LDL-C	Low-density lipoprotein cholesterol
NASCET	North American Symptomatic Carotid Endarterectomy Trial
OSI	Oscillatory shear index
OSS	Oscillatory shear stress
ROS	Reactive oxygen species
VSMC	Vascular smooth muscle cells
WSS	Wall shear stress
